# Heat-Shock Protein 27 (HSPB1) Is Upregulated and Phosphorylated in Human Platelets during ST-Elevation Myocardial Infarction

**DOI:** 10.3390/ijms20235968

**Published:** 2019-11-27

**Authors:** Bjoern F. Kraemer, Hanna Mannell, Tobias Lamkemeyer, Mirita Franz-Wachtel, Stephan Lindemann

**Affiliations:** 1 Medizinische Klinik und Poliklinik I, Klinikum der Universität München, Marchioninistrasse 15, 81377 Munich, Germany; bjoern.kraemer@klinik-ebe.de; 2 Walter Brendel Centre of Experimental Medicine, University Hospital, Ludwig-Maximilians-University, Marchioninistr. 27, 81377 Munich, Germany; 3 Biomedical Center, Ludwig-Maximilians-University, Großhaderner Str. 9, 82152 Planegg, Germany; 4 Cluster of Excellence Cologne (CEDAD), Mass Spectrometry Facility at the Institute for Genetics, University of Köln, Josef-Stelzmann-Str. 26, 50931 Köln, Germany; 5 Proteasome Center Tuebingen, University of Tuebingen, Auf der Morgenstelle 15, 72076 Tübingen, Germany; 6 FB 20–Medizin, Philipps Universität Marburg, Baldingerstraße, 35032 Marburg, Germany; 7 Medizinische Klinik II, Klinikum Warburg, Hüffertstr. 50, 34414 Warburg, Germany; 8 Medizinische Klinik und Poliklinik III, Universitätsklinikum Tübingen, Otfried-Muller-Str. 10, 72076 Tübingen, Germany

**Keywords:** platelets, myocardial infarction, HSP27, heat-shock proteins

## Abstract

Heat-shock proteins are a family of proteins which are upregulated in response to stress stimuli including inflammation, oxidative stress, or ischemia. Protective functions of heat-shock proteins have been studied in vascular disease models, and malfunction of heat-shock proteins is associated with vascular disease development. Heat-shock proteins however have not been investigated in human platelets during acute myocardial infarction ex vivo. Using two-dimensional electrophoresis and immunoblotting, we observed that heat-shock protein 27 (HSPB1) levels and phosphorylation are significantly increased in platelets of twelve patients with myocardial infarction compared to patients with nonischemic chest pain (6.4 ± 1.0-fold versus 1.0 ± 0.9-fold and 5.9 ± 1.8-fold versus 1.0 ± 0.8-fold; *p* < 0.05). HSP27 (HSPB1) showed a distinct and characteristic intracellular translocation from the cytoskeletal fraction into the membrane fraction of platelets during acute myocardial infarction that did not occur in the control group. In this study, we could demonstrate for the first time that HSP27 (HSPB1) is upregulated and phosphorylated in human platelets during myocardial infarction on a cellular level ex vivo with a characteristic intracellular translocation pattern. This HSP27 (HSPB1) phenotype in platelets could thus represent a measurable stress response in myocardial infarction and potentially other acute ischemic events.

## 1. Introduction

Heat-shock proteins (HSPs) are expressed in many cells of the cardiovascular system such as endothelial cells, cardiac muscle cells, monocytes, and platelets. They are upregulated in response to inflammation, oxidative stress, or ischemia [1] and protect cells against extracellular stress factors. In order to protect the cell against stress stimuli, heat-shock proteins function as chaperone proteins, stabilize the cytoskeleton [2], and prevent apoptosis [3,4,5]. Functions of HSP27 (HSPB1) are modulated by phosphorylation, although the precise mechanisms and functions of HSP27 regulation are not fully understood. Previous reports imply that phosphorylation of HSP27 appears to reflect cell protective functions [2,3,6] and seems to be the initiating step prior to intracellular translocation enabling HSP27 to interact with the cytoskeleton [7]. Increased phosphorylation of HSP27 has been observed during different modes of platelet activation [8,9,10] or cardiovascular risk conditions such as diabetes mellitus [11]. HSP27 directly interacts with the actin cytoskeleton and thus downregulates inflammatory cell migration [12]. Other heat-shock proteins such as HSP70 and HSP90 are also actively involved in integrin-mediated platelet adhesion [13], and HSP90 associates with the platelet glycoprotein Ib-IX-V complex [14]. Inactivation of heat-shock proteins has been associated with progression of atherosclerosis and acute vascular events. Increased expression or release of heat-shock proteins further seem to reflect systemic stress responses or ischemia [15]. Oxidized low density lipoprotein (LDL) molecules, for example, induce expression of heat-shock proteins [16], whereas decreased expression of protective HSPs was found in atherosclerotic vessel areas [17,18]. In patients with acute coronary syndrome, increased plasma levels of HSP27 and HSP70 were detected, although the source of HSP27 remained unknown [19,20,21]. Phosphorylated HSP27 is further released from platelets in patients with diabetes, which is a major risk factor for vascular disease [11].

In this work, we demonstrate for the first time that HSP27 (HSPB1) is upregulated and phosphorylated in platelets of patients with ST-elevation myocardial infarction compared to control patients with nonischemic chest pain. HSP27 in platelets showed a distinct intracellular translocation pattern that was not observed in the control group. 

## 2. Results

### 2.1. HSP27 Is Upregulated in Human Platelets during Myocardial Infarction

**Figure 1:**
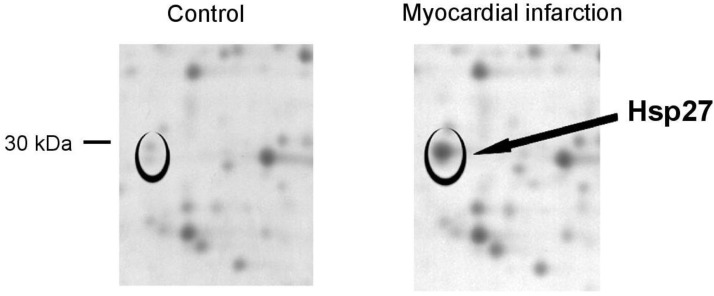
Heat-shock protein 27 (HSP27) is upregulated in platelets during myocardial infarction. Two-dimensional electrophoresis shows a protein spot that was upregulated in patients with ST-elevation myocardial infarction (black circles), which was identified as heat-shock protein 27 (HSP27) by mass spectrometry. Images show a representative sample of a patient with myocardial infarction and a matched control patient, which are representative of twelve independent patient pairs. Protein spots are visualized in a 2-fold magnification.

Protein spots that showed differential regulation in platelets during myocardial infarction in two-dimensional gel electrophoresis were sampled and analyzed by mass spectrometry. Figure 1 shows a protein that was consistently upregulated in platelets during myocardial infarction. We identified this protein as heat-shock protein 27 (HSP27) by amino acid sequencing in mass spectrometry. Increased HSP27 levels were observed in twelve patients with myocardial infarction compared to control samples.

### 2.2. HSP27 Is Upregulated and Phosphorylated in Human Platelets during Myocardial Infarction

**Figure 2:**
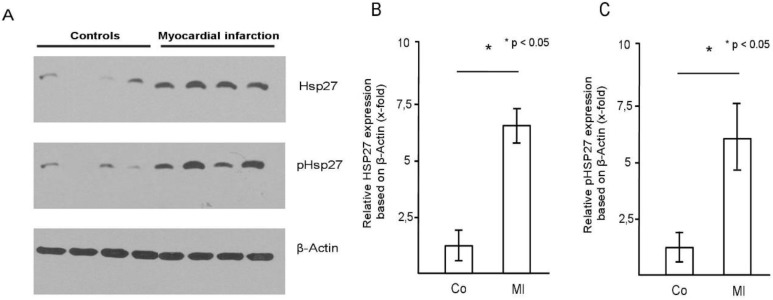
HSP27 is upregulated and phosphorylated in platelets during myocardial infarction. (**A**) HSP27 levels and phosphorylation (pHSP27) in four representative patients with nonischemic chest pain (controls, lanes 1–4) and four patients with myocardial infarction (MI) (lanes 5–8) are shown. β-actin served as loading control. (**B**) Quantitative analysis of HSP27 levels and phosphorylation (**C**) in the group of patients with myocardial infarction (MI) (*n* = 12) compared to controls (*n* = 12); * *p* < 0.05. Mean HSP27 and phospho-HSP27 to actin ratio for the control group was set as 1. The blot is representative of 12 independent patient pairs.

Increased HSP27 levels in platelets of patients with myocardial infarction compared to controls were confirmed by immunoblotting (Figure 2A). Detection of phosphorylated HSP27 (pHSP27) with a specific antibody also showed increased phosphorylation of HSP27 in myocardial infarction (Figure 2A). Four representative, independent patient pairs are shown that illustrate the increased HSP27 levels and phosphorylation in patients with myocardial infarction. Band densitometry of the Western blot results (*n* = 12 in each group) showed a significant increase of HSP27 protein and HSP27 phosphorylation in platelets from patients with myocardial infarction (MI) compared to controls (6.4 ± 1.0-fold versus 1.0 ± 0.9 and 5.9 ± 1.8-fold versus 1.0 ± 0.8; *p* < 0.05) (Figure 2B,C).

### 2.3. HSP27 Levels Are Increased by Thrombin Stimulation

**Figure 3:**
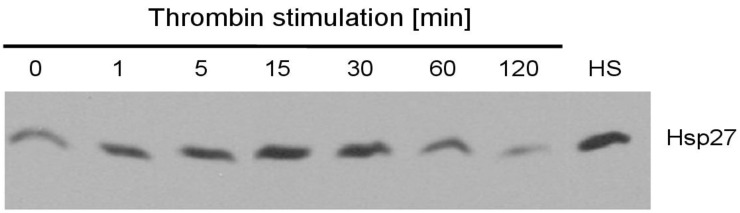
Thrombin activation induces upregulation of HSP27 in platelets. Platelets were stimulated with thrombin (0.5 U/mL) for 1, 5, 15, 30, 60, and 120 min, and HSP27 levels were quantified by immunoblotting. Heat activation at 42 °C for 10 min (HS) served as positive control. The immunoblot time course for HSP27 levels is representative of 3 independent experiments.

Platelets were stimulated with thrombin (0.5 U/L) for up to two hours, and HSP27 levels were quantified by immunoblotting. After thrombin stimulation, we observed a gradual increase of HSP27 with a peak value at 30 min, which declined with longer stimulation (Figure 3). Heat-shock treatment of platelets (HS, 42 °C, 10 min) induced a robust upregulation of HSP27 and served as a positive control. 

### 2.4. HSP27 Translocates from the Cytoskeletal into the Membrane Fraction of Platelets during Myocardial Infarction

**Figure 4:**
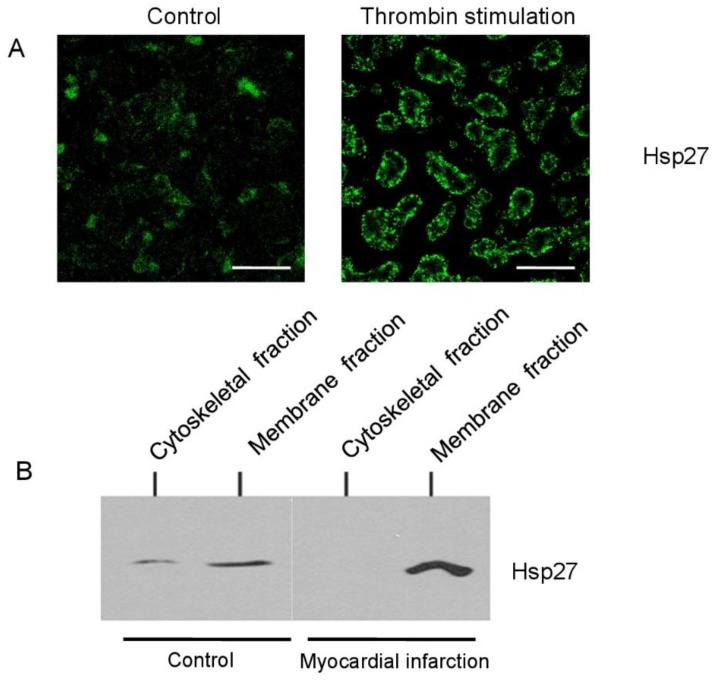
HSP27 translocates into the membrane fraction of platelets during myocardial infarction. (**A**) Confocal microscopy of resting (left) and thrombin activated platelets (0.5 U/L) (right) illustrates the characteristic translocation of HSP27 into the cell membrane of platelets with activation; scale bars represent 5 μm. (**B**) HSP27 distribution in platelets was further quantified in the membrane and cytoskeletal fraction of platelets from patients with nonischemic chest pain (controls, lanes 1–2) and patients with myocardial infarction (lanes 3–4). The immunoblot is representative of twelve independent patient pairs.

Thrombin stimulation of platelets induced an intracellular translocation of HSP27 from the cytoskeletal into the membrane-associated fraction of platelets as illustrated by confocal microscopy (Figure 4A). To quantify intracellular HSP27 distribution, platelet lysates were separated into the cytoskeletal and plasma membrane fraction by stepwise ultracentrifugation. Figure 4B shows a representative Western blot of a patient pair from the study population. Resting control platelets only showed small HSP27 levels in both the membrane and cytoskeletal fraction. During myocardial infarction, HSP27 was found at increased levels in the membrane fraction, which was not observed in control patients. 

## 3. Discussion

In the present study, we used two-dimensional electrophoresis to analyze proteomic changes of platelets from patients with acute myocardial infarction and nonischemic chest pain and observed increased phosphorylation (pHSP27) and upregulation of HSP27 in platelets during myocardial infarction. So far, intracellular modulation of HSP27 has not been studied in human platelets during myocardial infarction ex vivo. 

Patients with ST-elevation myocardial infarction were treated with aspirin immediately after diagnosis, and additional loading with ADP receptor antagonists such as clopidogrel was initiated immediately after blood was drawn. All control patients were also treated with aspirin by the time of blood sampling due to acute onset chest pain and suspected coronary artery disease. Therefore, there was no difference in antiplatelet therapy between groups at the time of blood sampling to make sure that the observed effects can be attributed to the acute vascular event and not to drug-related effects.

Phosphorylation of HSP27 seems to indicate that the protein is in a cell protective, anti-oxidative, and antiapoptotic functional state [2,3,22]. The role of heat-shock proteins in inflammation and its anti-oxidative capacity is of paramount interest, and animal studies have been conducted to investigate the use of heat-shock proteins in anti-atherosclerotic therapy [8,18,23]. Heat-shock proteins are activated in response to oxidized LDL and mediate an anti-inflammatory response through release of Interleukin 10 (IL-10) [24], through increased levels of gluthathione [25], and through inactivation of Nuclear Factor kappa B (NFκB) [26]. Low-density lipoproteins in return induce dephosphorylation of HSP27 [27], which alters anti-oxidative functions of HSP27. Malfunction or downregulation of heat-shock proteins has been associated with vascular disease progression and atherosclerosis [17,18,21]. Furthermore, decreased expression of heat-shock proteins was observed in atherosclerotic vascular areas and vulnerable plaques, which could be a sign of defective local anti-oxidative protection [1,2]. 

Heat-shock proteins were found in increased concentrations in the plasma of patients with acute myocardial infarction or coronary artery disease although the source remained unknown [8,28,29], and HSP27 levels were increased after global ischemia in coronary sinus blood samples of patients after surgical aortic clamping [15]. Altogether, heat-shock protein expression appears to be increased in response to vascular ischemia and compromised function of heat-shock proteins favors vascular disease development. Release of phosphorylated HSP27 from platelets was found in patients with diabetes who are at great risk for vascular disease [11]. 

Although we found a robust HSP27 phenotype in platelets during myocardial infarction, it remains to be determined if this is a result of the ischemic process or whether these changes occur prior to the acute event. Further studies suggest that HSP27 also regulates the cytoskeleton and that phosphorylation of HSP27 is a step that immediately precedes the association of HSP27 with the activated cytoskeleton of platelets [7] to stabilize actin fibers. 

In this study, we consistently observed a distinct intracellular redistribution pattern from the cytoskeletal into the membrane-associated protein fraction in platelets during myocardial infarction, which was not observed in patients with nonischemic chest pain. Previous observations demonstrate that HSP27 translocates between cellular compartments in platelets after thrombin stimulation [7]. Supporting these observations, we could demonstrate that thrombin induces an upregulation of HSP27 with a peak value at 30 min and a characteristic translocation into the platelet membrane in confocal microscopy. In addition, phosphorylation of HSP27 in platelets has been closely correlated with platelet dense granule secretion [8,9,10], which is another characteristic of platelet activation. Plasma levels of phosphorylated HSP27 correlated closely with platelet aggregation in patients with diabetes; however, it remains unclear if this finding is functionally connected [11]. 

Since HSP27 directly associates with the cytoskeleton to execute signaling functions, intracellular translocation of HSP27 during cellular activation or in response to stress signals appears to make sense physiologically. 

Others have reported that HSP27 interacts with the cytoskeleton through actin fiber stabilization, which has also been associated with decreased migratory capacity of inflammatory cells and anti-inflammatory functions [12]. We have previously shown that platelets also gain migratory capacity in response to inflammatory signals and that platelets can transmigrate into the vessel wall [30]. The role of heat-shock proteins in this context however is still unknown. 

Besides direct regulatory effects on a cytoskeletal level, HSP27 controls translation factors such as eIF4F through binding of eIF4G, which then results in inhibition of translation [31]. Likely HSP27 prevents production of inflammatory proteins as part of an anti-inflammatory response that way. The above findings support the hypothesis and give reason to believe that phosphorylation of HSP27 may be a step that enables HSP27 to execute its cell protective effects. 

An elegant study by Liu and colleagues demonstrates increased levels of CCL2 (Chemokine CC-motif ligand 2) in plasma, platelets, and thrombus material of patients with myocardial infarction. Activation of platelets with CCL2 in return resulted in increased phosphorylation of HSP27 and other signaling proteins [32]. 

It is tempting to speculate that increased phosphorylation of HSP27 in platelets from patients with myocardial infarction could be a sign of an ischemic stress response. We do not know however if this phenotype is a direct result of the acute vascular events or a consequence of platelet activation. The question whether heat-shock protein phosphorylation is an anti-inflammatory reaction in platelets while unphosphorylated HSP27 may contribute to accelerated aggregation or if it is a passive result of the stress stimulus remains to be determined in future studies. Further research will additionally be necessary to investigate the regulatory mechanisms of HSP27 protein and its phosphorylation during myocardial infarction. 

In summary, we observed a significant increase of HSP27 (HSPB1) protein levels and phosphorylation of HSP27 in human platelets during myocardial infarction compared to matched controls with nonischemic chest pain in this study. Supporting previous observations in platelets, a characteristic intracellular translocation of HSP27 from the cytoskeletal into the membrane associated protein fraction was also observed during myocardial infarction. This platelet phenotype was distinctly different in platelets from patients with myocardial infarction compared to controls. It will be interesting to extend the observed characteristics of the HSP27 phenotype in platelets during myocardial infarction to other acute vascular events such as ischemic stroke or peripheral artery disease. 

## 4. Material and Methods

### 4.1. Study Design

Twelve patients with acute ST-elevation myocardial infarction (mean age 62 years) and obstruction of a proximal left dominant or right coronary artery as well as twelve age-matched control patients with acute onset typical chest pain but rule-out for acute myocardial infarction by electrocardiogram (ECG) and cardiac enzymes were enrolled in the study. Due to suspected coronary artery disease, control patients underwent coronary angiography in which coronary artery disease was ruled out. According to guideline recommendations, patients with ST-elevation myocardial infarction were treated with aspirin immediately after diagnosis. Blood for this study was drawn in the cath lab before other antiplatelet agents such as clopidogrel or tirofiban were administered for acute myocardial infarction. All control patients were also on aspirin treatment by the time of blood sampling. Written informed consent was obtained from all patients. The study was approved by the local ethics committee of the University of Tübingen (No. 264/2007BO2, approved 25 October 2007) in accordance with the Declaration of Helsinki. Blood was drawn on admission after indication for coronary angiography had been established and blood samples were processed immediately. 

### 4.2. Platelet Isolation

Platelets were isolated according to standard protocols in our lab as previously described [30,33]. For some experiments, platelets were stimulated with thrombin (Sigma-Aldrich, Taufkirchen, Germany) 0.1 U/mL for different time points or were heat-activated at 42 °C for 10 min prior to lysis. A total of 1 × 10^9^ platelets was lysed in 100 µL of CHAPS lysis buffer (8 M Urea, 4% CHAPS, and 2% DTT) and stored for two-dimensional gel analysis and immunoblotting at −20 °C.

### 4.3. Fractionation of Cytoskeleletal and Membrane Proteins of Platelets

Freshly isolated platelets (1 × 10^9^) were lysed in 1 mL Triton X-lysis buffer (containing complete protease inhibitor, Roche, Penzberg, Germany) for 10 min on ice. The pellet containing the cytoskeletal cell fraction was collected after centrifugation at 15,600× *g* for 4 min after supernatants had been carefully removed. Supernatants were centrifugated again at 100,000× *g*, and pellets containing the membrane fraction were collected. Both fractions were resuspended in SDS loading buffer before electrophoresis.

### 4.4. Two-Dimensional Electrophoresis

Platelets from patients with myocardial infarction and nonischemic chest pain were lysed in 100 µL of CHAPS lysis buffer (8 M Urea, 4% CHAPS, and 2% DTT) and purified using a 2D clean-up kit (GE Healthcare, Freiburg, Germany) according to the manufacturer’s instructions. Comparative 2D gel analysis of the proteomes was performed as described previously with slight modifications [34,35]. First dimension isoelectric focusing was performed using a Protean IEF Cell focusing unit (BioRad, Hercules, CA, USA) with pH 3–10 NL gel strips (11 cm, GE Healthcare, for some subsequent gels pH 4–7). For the second dimension equilibrated (5.7 M Dithiotreitol and 1.5 M Iodoacetamide) gel strips were applied to 12% polyacrylamide gels. Proteins were stained with Lava purple fluorescent staining (Fluorotechnics, Sydney, Australia). Protein spots of interest were excised manually from the gels, digested with trypsin. and analyzed by LC ESI-MS/MS (Applied Biosystems/MDS Sciex, Darmstadt, Germany) as described previously [35]. 

### 4.5. Immunoblotting

Platelets lysed in Laemmli buffer were separated by SDS PAGE gel electrophoresis and probed for HSP27, phospho-HSP27, and β-actin. After protein transfer to a nitrocellulose membrane, membranes were blocked in 5% nonfat milk in Tris-buffered saline with Tween-20 (TBS-T, Sigma-Aldrich, Taufkirchen, Germany) for 1 h. Afterwards, primary monoclonal antibody to HSP27 or phospho-HSP27 (1:1000, Santa Cruz Biotechnologies, Dallas, TX, USA) were added and incubated overnight at 4 °C with constant agitation. Membranes were washed in TBS-T repeatedly, and HRP-conjugated secondary antibody (1:10000) (GE Healthcare, Freiburg, Germany) was added for 1 h at room temperature. After washing, chemiluminescent substrate (ECL reagent, Amersham Biosciences, Munich, Germany) was added for 1–5 min and bands were visualized on plain film. β-Actin served as loading control (1:000, rabbit anti-human β-Actin, Cell Signaling Technology, Dallas, TX, USA). HSP27 and phospho-HSP27 levels were quantified by band densitometry analysis with the help of HSP27- and pHSP27-to-actin ratios. Mean HSP27- and pHSP27-to-actin ratios for the myocardial infarction group were set as 1 and relative increases in HSP27 and pHSP27 levels in the control group were compared accordingly. Relative HSP27 and pHSP27 levels between groups based on actin loading are shown.

### 4.6. Confocal Microscopy

Immunofluorescence staining was performed as previously described [30]. In brief, washed platelets were allowed to adhere to a fibrinogen surface (50 μg/mL) on a chamber slide for 20 min and were then fixed with paraformaldehyde (2%) and permeabilized with Triton-X-100 (TX-100, 0.025%). The adherent platelets were washed and blocked with 2% bovine serum albumin for 30 min followed by incubation with the primary antibody for 2 h at room temperature. Primary antibody against HSP27 (Santa Cruz Biotechnologies, Dallas, TX, USA) was used in a 1:100 dilution in TBS with 1% BSA (Bovine Serum Albumine). Slides were then washed and incubated with an Alexa488-linked secondary antibody (Dianova, Hamburg, Germany) for 1 h. Confocal microscopy was performed using a Zeiss LSM 5 EXCITER confocal laser scanning microscope (Carl Zeiss Micro Imaging, Jena, Germany).

### 4.7. Statistical Analysis

All data are presented as means ± SEM. Statistical analyses were performed with Sigma Plot 10.0. For comparisons between two groups of normal distributed data, the student´s t-test was used. For the comparison of two groups without normal distributed data, a rank-sum test was performed. For multiple comparisons between groups of normal distributed data, the one-way analysis of variance (1-way ANOVA) was used. Differences were considered significant at an error probability level of *p* < 0.05.

## 5. Conclusions

We observed a significant increase of HSP27 (HSPB1) levels and phosphorylation of HSP27 in platelets during myocardial infarction compared to matched controls with nonischemic chest pain in this study. Supporting previous observations in platelets, a characteristic intracellular translocation of HSP27 from the cytoskeletal into the membrane-associated protein fraction was also observed during myocardial infarction. This platelet phenotype was distinctly different in platelets from patients with myocardial infarction compared to controls. It will be interesting to extend the observed characteristics of the HSP27 phenotype in platelets during myocardial infarction to other acute vascular events such as ischemic stroke or peripheral artery disease.
